# A novel chalcone derivative has antitumor activity in melanoma by inducing DNA damage through the upregulation of ROS products

**DOI:** 10.1186/s12935-020-1114-5

**Published:** 2020-01-30

**Authors:** Keke Li, Shuang Zhao, Jing Long, Juan Su, Lisha Wu, Juan Tao, Jianda Zhou, JiangLin Zhang, Xiang Chen, Cong Peng

**Affiliations:** 1 The Department of Dermatology, Xiangya Hospital, Central South University, 87 Xiangya Road, Changsha, Hunan China; 2 Hunan Key Laboratory of Skin Cancer and Psoriasis, Xiangya Hospital, Central South University, Changsha, Hunan China; 3 Hunan Engineering Research Center of Skin Health and Disease, Xiangya Hospital, Central South University, Changsha, Hunan China; 4 Department of Dermatology, Affiliated Union Hospital, Tongji Medical College, Huazhong University of Science and Technology, Wuhan, China; 5 Department of Plastic Surgery of Third Xiangya Hospital, Central South University, Changsha, China

**Keywords:** Chalcone, DNA damage, Melanoma, P53, ROS (reactive oxygen species)

## Abstract

**Background:**

Melanoma is one of the most aggressive tumors with the remarkable characteristic of resistance to traditional chemotherapy and radiotherapy. Although targeted therapy and immunotherapy benefit advanced melanoma patient treatment, BRAFi (BRAF inhibitor) resistance and the lower response rates or severe side effects of immunotherapy have been observed, therefore, it is necessary to develop novel inhibitors for melanoma treatment.

**Methods:**

We detected the cell proliferation of lj-1-59 in different melanoma cells by CCK 8 and colony formation assay. To further explore the mechanisms of lj-1-59 in melanoma, we performed RNA sequencing to discover the pathway of differential gene enrichment. Western blot and Q-RT-PCR were confirmed to study the function of lj-1-59 in melanoma.

**Results:**

We found that lj-1-59 inhibits melanoma cell proliferation in vitro and in vivo, induces cell cycle arrest at the G2/M phase and promotes apoptosis in melanoma cell lines. Furthermore, RNA-Seq was performed to study alterations in gene expression profiles after treatment with lj-1-59 in melanoma cells, revealing that this compound regulates various pathways, such as DNA replication, P53, apoptosis and the cell cycle. Additionally, we validated the effect of lj-1-59 on key gene expression alterations by Q-RT-PCR. Our findings showed that lj-1-59 significantly increases ROS (reactive oxygen species) products, leading to DNA toxicity in melanoma cell lines. Moreover, lj-1-59 increases ROS levels in BRAFi -resistant melanoma cells, leading to DNA damage, which caused G2/M phase arrest and apoptosis.

**Conclusions:**

Taken together, we found that lj-1-59 treatment inhibits melanoma cell growth by inducing apoptosis and DNA damage through increased ROS levels, suggesting that this compound is a potential therapeutic drug for melanoma treatment.

## Background

Cutaneous melanoma is the most aggressive type of skin cancer [1, 2], and its incidence and mortality is increasing annually worldwide [3, 4]. In 2017, there were 87,110 patients diagnosed with melanoma, and 9730 cases were proposed deaths from malignant melanoma [5]. Although primary melanoma has been cured by surgical therapy, melanoma cells from the primary tumor break through the basement membrane in the early stage and invade the lymphatics or vasculature, leading to the formation of metastatic lesions in distant organs, including the lungs, brain and liver [6], resulting in a 5-year survival rate of less than 10% [7]. The risk factors of melanoma development involve genetic and environmental effects [8]. NGS (next generation sequencing) studies identified somatic mutations that generate a landscape of melanoma somatic mutations, including *BRAF, NRAS, NF1, PTEN, CDKN2A* and *TP53*, as driving mutations or potential tumor suppressors and oncogenes [9].

The BRAFV600E mutation, as a frequent somatic mutation, occurs in approximately 60% of melanomas, causing the constitutive activation of the mitogen-activated protein kinase (MAPK) pathway [10]. Therefore, the BRAFV600E mutation acts as a pivotal oncogenic driver gene in melanoma, leading to the development of targeted BRAF kinase inhibitors. PLX4720 has been developed as a BRAFV600E inhibitor versus BRAF wild-type tumors in vivo based on a structural analog, which was approved for the treatment of advanced metastatic melanoma patients with BRAFV600E mutation expression [11]. MEK inhibitors, such as trametinib, have been approved for the treatment of advanced melanoma patients carrying the BRAF mutation [12]. In a clinical trial, a 22% response and 4.8-month median progression-free survival were observed in patients with metastatic melanoma expressing BRAFV600E/K after trametinib therapy compared with chemotherapy [13]. Although BRAFi significantly benefits clinical responses and promotes advanced melanoma patient survival, drug resistance and relapse can develop over several months of treatment [14].

The development of novel immunotherapies, such as anti-CTLA4 and anti-PD-1, has significantly improved melanoma patient outcomes [15, 16]. CTLA-4 is an immune checkpoint receptor [17] and the first receptor targeted by a clinical therapeutic antibody (ipilimumab) approved by the FDA in 2011 [18]. PD-1 is another T cell inhibitory receptor that exerts immune suppression through PD-1 ligand (PD-L1) [19]. Blockade of PD-1 or PD-L1 with therapeutic antibodies benefits the activation of tumor antigen-specific T cells but does not affect autoreactive T cells. These recent immunotherapies dramatically reduce tumor burden and benefit advanced melanoma patient overall survival [20]. However, the clinical response is approximately 20–30% [21], and at the same time, these therapies have some fatal side effects. Therefore, it is necessary to develop novel inhibitors for melanoma treatment.

Chalcone is one of the numerous natural compounds that is widely found in fruits, vegetables and tea [22, 23]. Chalcone has various biological activities, including anti-inflammatory, antibacterial and antioxidant activities [24, 25]. Chalcone has been shown to have a skeletal structure for antitumor treatment, such as lung cancer, colorectal cancer, liver cancer and breast cancer [26–28]. Therefore, chalcone derivatives have been widely studied for antitumor pharmacological activities.

In this study, we found a chalcone derivative, lj-1-59 ((E)-1-(3-hydroxyphenyl)-3-(3,4,5-trimethoxyphenyl)prop-2-en-1-one) synthesized from 3,4,5-trimethoxybenzaldehyde and 3′-hydroxyacetophenone through Claisen-Schmidt reaction in our lab, significantly inhibits melanoma cell growth in vitro and in vivo. Furthermore, this compound significantly increases ROS products as a consequence of induced apoptosis and G2/M phase arrest through ROS-mediated DNA damage, resulting in the activation of ATM, ATR and H2AX, suggesting that this compound is a promising medicine for melanoma treatment.

## Methods

### Chemicals

**Fig. 1:**
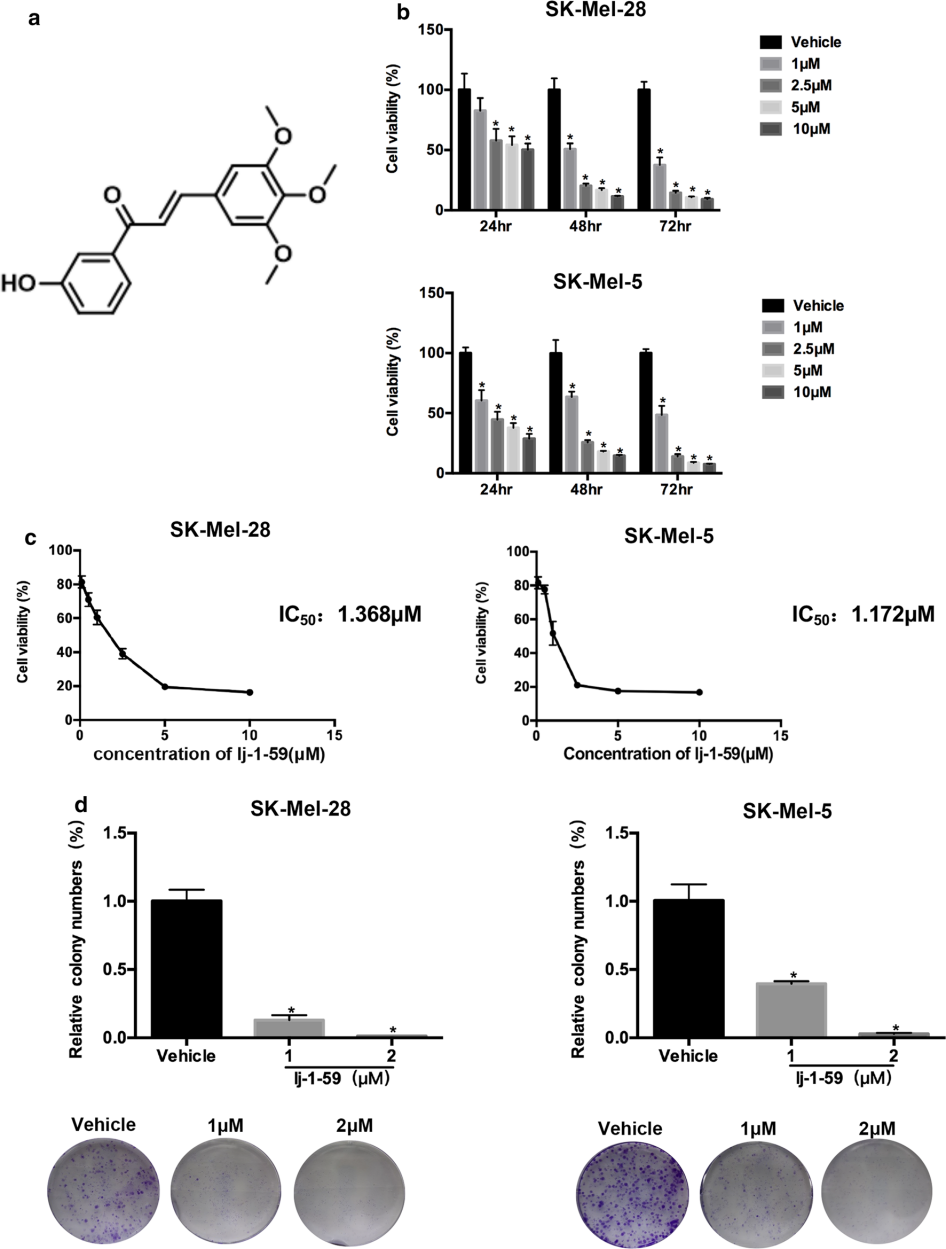
lj-1-59 inhibits the proliferation of human melanoma cells. **a** Structure of lj-1-59. **b** SK-Mel-28 (upper panel) and SK-Mel-5 (lower panel) were prepared in 96-well plates. The cells were treated with increasing dose lj-1-59 for 0-72 h. Cell viability was determined by CCK-8 assay. The results represent the means (n = 6) ± SD. Significant differences were evaluated using Student’s t-test, and an asterisk (*) indicates a significant difference (p < 0.05). **c** The IC_50_ values of lj-1-59 in SK-Mel-28 (left panel) and SK-Mel-5 (right panel) cells were automatically calculated for 48 h by GraphPad Prism software. **d** SK-Mel-28 (left panel) and SK-Mel-5 (right panel) cells were prepared in 6-well plates. The cells were treated with increasing dose lj-1-59 for 24 h. After 2 weeks, the number of colonies was assessed and quantified as described in “Methods”. The data represent the mean (n = 4) ± SD, and an asterisk (*) indicates a significant difference (p < 0.05, Student’s t-test)

lj-1-59((E)-1-(3-hydroxyphenyl)-3-(3,4,5-trimethoxyphenyl)prop-2-en-1-one) (Fig. 1a) was synthesized as follow: To a cold solution of NaOMe (216 mg) in MeOH (4 mL) was added 3,4,5-trimethoxybenzaldehyde (200 mg, 1 mmol) and 3′-hydroxyacetophenone (139 mg, 1 mmol), stirred for 48 h at room temperature. Concentrated, added 3 mL H_2_O, washed with Et_2_O three times, added 12 N HCl until pH = 1. Extracted with EtOAc three times, The organic extracts was washed with brine, dried over Na_2_SO_4_, filtered, and concentrated, recrystallized from EtOH/H_2_O to get the product (236 mg, 75%). ^1^H NMR (500 MHz, DMSO) δ 9.82 (s, 1H), 7.83 (d, *J* = 15.6 Hz, 1H), 7.72–7.60 (m, 2H), 7.48–7.46 (m, 1H), 7.39 (t, *J* = 7.9 Hz, 1H), 7.23 (s, 2H), 7.07 (dd, *J* = 8.1, 1.3 Hz, 1H), 3.87 (s, 6H), 3.72 (s, 3H); ^13^C NMR (125 MHz, DMSO) δ 189.6, 158.2, 153.6, 144.8, 140.2, 139.6, 130.7, 130.2, 121.9, 120.7, 120.1, 115.1, 107.0, 60.6, 56.6. lj-1-59 was diluted to 50 mM in DMSO and stored at − 20 °C.

### Cell lines and culture

The human melanoma cell lines A375, SK-Mel-5, and SK-Mel-28 were purchased from the ATCC (Manassas, VA, USA), and the BRAFi-resistant cell line, called RA, was generated as described in a previous study [9, 29]. JB6 mouse skin epidermal cell line, BJ human skin fibroblast cell line,PIG1 human melanocyte cell line, H9C2 human heart myoblast cell line were purchased from the ATCC. Both cell lines were cultured at 37 °C in DMEM (10% FBS, 1% penicillin–streptomycin). RA cells were maintained in culture with 2 µM vemurafenib (PLX4032), and the drug was removed 1 week before use.

### Cytotoxicity assay (CCK-8)

The cells were seeded onto 96-well plates (2000 cells/well) and treated with lj-1-59 for 24, 48 and 72 h, and the cells were assessed using the CCK-8 assay (Selleckchem, Houston, USA). We used the OD value of 48 h after lj-1-59 treated to calculate the IC_50_ value. The IC_50_ values were automatically calculated by using GraphPad Prism software.

### Colony formation assay

The cells were seeded onto 6-well plates (1500 cells/well) and treated with lj-1-59 or DMSO (Vehicle) for 24 h under standard culture conditions. Next, the medium was replaced, and the cells were cultured for approximately 14 days in normal medium. The cells were then stained with crystal violet after fixation with 4% paraformaldehyde.

### Cell apoptosis and cell cycle assay

The cells were treated with lj-1-59 or DMSO (Vehicle) for 48 h and then stained with Annexin V-FITC/PI (BD Biosciences, New Jersey, USA). Cell apoptosis was measured by flow cytometry and analyzed by using FlowJo software. For the cell cycle assays, the cells after treatment with lj-1-59, fixed in cold 70% ethanol and stained with PI for 15 min at room temperature. Cell cycle was measured by flow cytometry and analyzed by using ModFit software. In another experiment, the cells were treated with 10 mmol/L *N*-acetylcysteine (NAC) (Beyotime, China) and 5 μM lj-1-59 for 48 h, then stained with Annexin V-FITC/PI or PI.

### Western blot analysis

The cells were lysed with RIPA buffer containing protease and phosphatase inhibitors (Selleckchem). For histone extraction, cells were lysed with NETN buffer containing protease and phosphatase inhibitors, and histones were extracted with HCL. The protein concentration was tested with a BCA kit, and appropriate amounts of protein were prepared for SDS-PAGE and then transferred to PVDF membrane (Millipore, MA, USA). The membranes were blocked for 1 h with 5% nonfat dry milk and then incubated with rabbit anti-p-ATM mAb (Ser1981; 1:1000; #5883; CST) and rabbit anti-p-ATR mAb (Ser428; 1:1000; #2853; CST), mouse anti-P53 (1:500; sc-47698; Santa Cruz) mAb, rabbit anti-ATR mAb (1:1000; #13934; CST), rabbit anti-ATM mAb (1:1000; #2873; CST), rabbit anti-PARP mAb (1:1000; #9532; CST), rabbit anti-Bcl2 mAb (1:1000; Cat. No. 12789-1-AP; Proteintech), rabbit anti-Bax mAb (1:1000; Cat. No. 50599-2-lg; Proteintech), rabbit anti-r-H2AX mAb (Ser139; 1:1000; #9718; CST), rabbit anti-P21 mAb (1:1000; Cat. No. 10355-1-AP; Proteintech), rabbit anti-H2AX mAb (1:500; D155226-0025; Sangon Biotech). Additionally, α-tubulin (1:1000; #5335; CST) and GAPDH (1:3000; Cat. No. 60004-1-lg; Proteintech) were used as loading controls. The results were imaged using a gel image analysis system (Bio-Rad, California, USA) according to the manufacturer’s instructions.

### Quantitative real-time PCR analysis

Total RNA was extracted from the cell samples using Trizol reagent (Invitrogen, California, USA) according to the manufacturer’s instructions. A reverse transcription reaction was performed using the SuperScript III First-Stand Synthesis System (Invitrogen, California, USA). The cDNA was amplified in SYBR Green qPCR mix (TOYOBO, Japan) and loaded onto the 7500 real-time PCR system (Applied Biosystems, MA, USA). GAPDH was used as an internal control. The primer sequences are as follows: h*SESN2*Fw5′tggctcatcaccaaggaacacatc3′; h*SESN2*Rv5′aggagagcgagtggcagtgg3′; h*ACTL8*Fw5′gcagcagagtgccttggatgag3′; h*ACTL8*Rv5′tctcgcaggactccacggattc3′; h*MCM3*Fw5′tcagacaccgccaggacatctc3′; h*MCM3*Rv5′caggtccacagtcttgctcatgc3′; h*MCM4*Fw5′cctcgcctggagtggacctg3′; h*MCM4*Rv5′gagtgccgtatgtcagtggtgaac3′; h*MCM2*Fw5′ggcgaggaggacgaggagatg3′; h*MCM2*Rv5′aagttcttgaagcggtggtggatc3′; h*MCM7*Fw5′ttggtaactgtgcgtggaatcgtc3′; h*MCM7*Rv5′ctggatcggctggtaggtctctg3′; h*CDKN1A*Fw5′agcgaccttcctcatccacc3′; h*CDKN1A*Rv5′aagacaactactcccagccccata3′; h*BBC3*Fw5′tctcctctcggtgctccttcact3′; h*BBC3*Rv5′acgtttggctcatttgctcttca3′; h*GADD45A*Fw5′ctcaagcagttactaaataca3′; and h*GADD45A*Rv5′cttcttcattttcacctctttcca3′.

### Measurement of ROS

The cells were seeded onto 1 × 10^6^ cells in 6-well plates and treated with 5 µM lj-1-59 for 0–6 h. In another experiment, the cells were pretreated with 5 mmol/L *N*-acetylcysteine (NAC) for 1 h, then cultured for 6 h with 5 μM lj-1-59. The medium was changed to serum-free medium, and the cells were incubated with DCFH-DA (Solarbio, China) for 30 min at 37 °C in the dark. DCFH-DA was deacetylated by intracellular esterase, which was oxidized by intracellular ROS to the fluorescent DCF. DCF fluorescence was detected using flow cytometer and analyzed by using FlowJo software.

### Immunofluorescence analysis

Cells (3 × 10^5^/well) were grown on coverslips, treated with lj-1-59 for 0–48 h, fixed with 4% paraformaldehyde for 15 min, and permeabilized with 0.5% Triton X-100 for 15 min. After blocking with 5% BSA. The cells were incubated with anti-γH2AX (Ser139; 1:100; #9718; CST) overnight at 4 °C. The next day, the cells were washed three times with PBS and incubated with secondary antibody for 1 h at room temperature. The cells were counterstained with DAPI and visualized by fluorescence microscopy.

### Immunohistochemistry

Tumors from nude mice were fixed and embedded in paraffin. The sections were baked at 65 °C for 2 h and treated with hydrogen peroxide after dehydrating in a series of graded alcohols. Antigen retrieval was performed by heat treatment in a pressure cooker in citrate buffer (pH = 6.0). The slides were blocked in goat serum for 1 h. Subsequently, the slides were incubated with Ki67 (1:400; ab16667; Abcam) at 4 °C overnight. The slides were incubated with a specific HRP-conjugated secondary antibody and stained with DAB. After PBS rinsing, the samples were counterstained in hematoxylin, dehydrated and mounted.

### Animal studies

Female BALB/c nude mice (5 weeks old) were purchased from the Central South University. Fifteen mice (18 g) were divided randomly into 3 groups, including the vehicle group (corn oil), the 20 mg/kg lj-1-59 group and the 40 mg/kg lj-1-59 group. Sk-Mel-5 cells (2 × 10^6^) were subcutaneously injected into the flanks of mice. When the tumors reached 50 mm^3^ or larger, each group of mice was injected through intraperitoneal injection with the corresponding drugs once a day for 2–3 weeks (approximately 16 times). The tumor size was measured using a caliper three times a week, and the tumor volume was calculated with the formula V = 1/2 (length × width^2^). When the tumors reached 1000 mm^3^, the mice were sacrificed, and the tumors were collected. The tumors were photographed. Furthermore, the tumor sections were immunostained.

### Statistical analysis methods

The significant differences between different groups was determined with ANOVA and Student’s t-test. A *p*-value < 0.05 was considered statistically significant. Statistical analysis was performed by GraphPad Prism.

## Results

### lj-1-59 blocks melanoma cell growth in vitro and in vivo

**Fig. 2:**
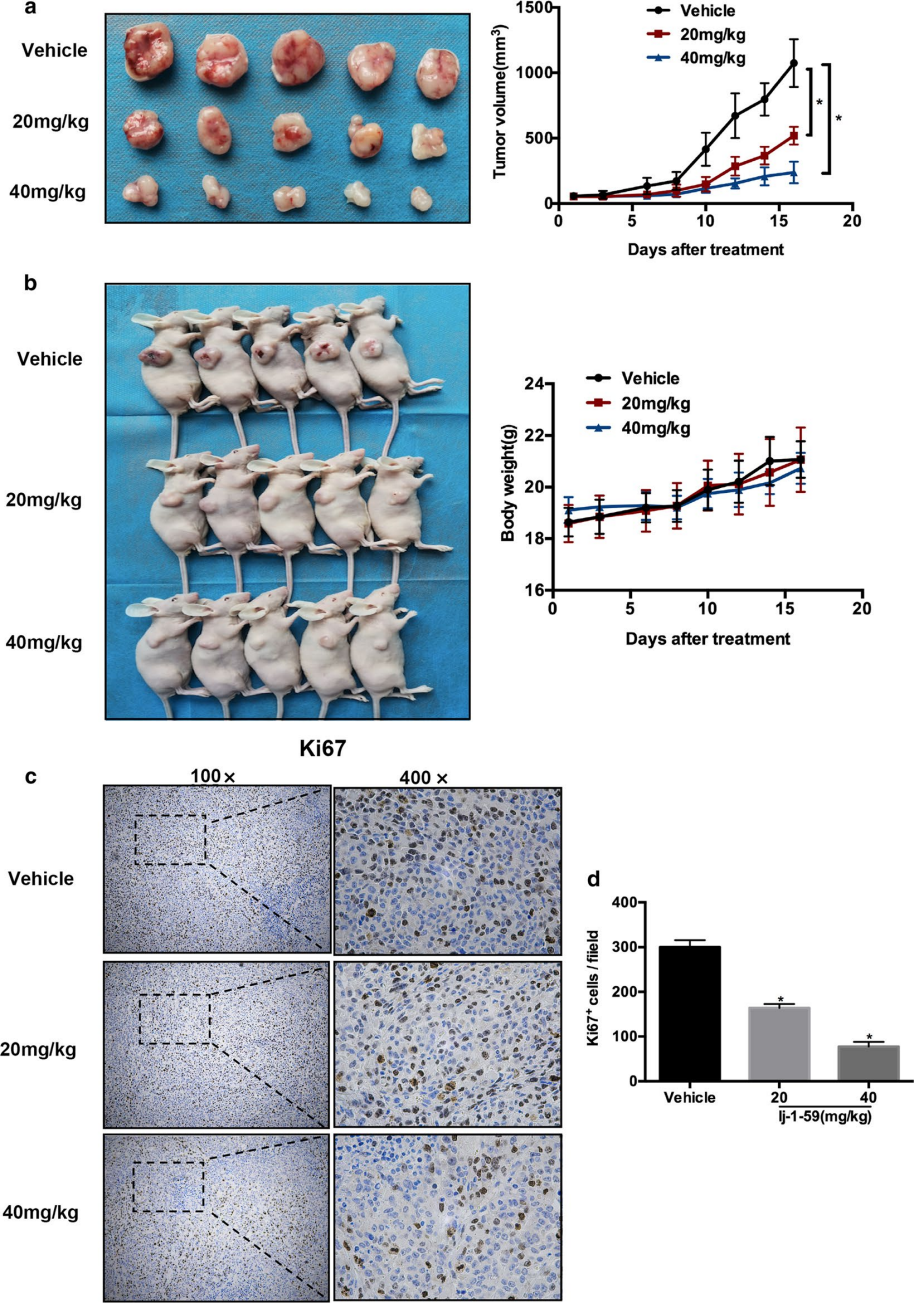
lj-1-59 suppresses xenograft tumor growth in vivo. **a** The tumor volume of nude mice. **b** The body weight of nude mice. The results in **a** and **b** are shown as the mean (n = 5) ± SD, and asterisk (*) indicates a significant difference (p < 0.05 one way ANOVA). **c** Representative images of IHC staining of Ki67 in tumor tissues. **d** Quantification of the Ki67 staining. Five images fields were analyzed per tumor slice. The results represent the means (n = 5) ± SD, and asterisk (*) indicates a significant difference (p < 0.05, Student’s t-test)

lj-1-59 is a novel chalcone derivative (Fig. 1a), and we determined the effect of lj-1-59 on melanoma cell growth. As shown in Fig. 1b and Additional file 1: Fig. S2a, the cell viability was significantly reduced after lj-1-59 treatment in various melanoma cells in a dose- and time-dependent manner. The IC_50_ values in SK-Mel-5, SK-Mel-28 and A375 were 1.172 µM, 1.368 µM and 2.002 µM, respectively, after 48 h lj-1-59 treatment (Fig. 1c and Additional file 1: Fig. S2a). Moreover, lj-1-59 treatment remarkably abrogated melanoma cell colony formation and growth on plates (Fig. 1d and Additional file 1: Fig. S2b). In addition, as shown in Additional file 1: Fig. S1, The IC_50_ values in PIG1 (human melanocyte cell line), JB6 (normal mouse skin epidermal cell line) and BJ (normal human skin fibroblast cell line) were 4.2 μM, 5.3 μM and 5.9 μM, respectively. IC_50_ values in H9C2 (normal human heart myoblast cells) was greater than 10 μM. These results suggested that the cytotoxicity of lj-1-59 was selective to melanoma cells. Taken together, these results confirmed that lj-1-59 inhibited the growth properties of human melanoma cells, including SK-Mel-5, SK-Mel-28 and A375. To study the effects of lj-1-59 on melanoma cell growth in vivo, we generated a melanoma cell xenograft mouse model. Consistent with previous results in vitro, lj-1-59 treatment reduced tumor burden at both low and high dosages (Fig. 2a, b) but did not affect body weight, indicating that this compound has low toxicity. Moreover, Ki67 staining was decreased in xenograft tissue after lj-1-59 treatment (Fig. 2c, d), suggesting that lj-1-59 significantly attenuates melanoma cell growth in vivo.

### lj-1-59 arrests the cell cycle at G2/M phase and induces apoptosis in melanoma cells

**Fig. 3:**
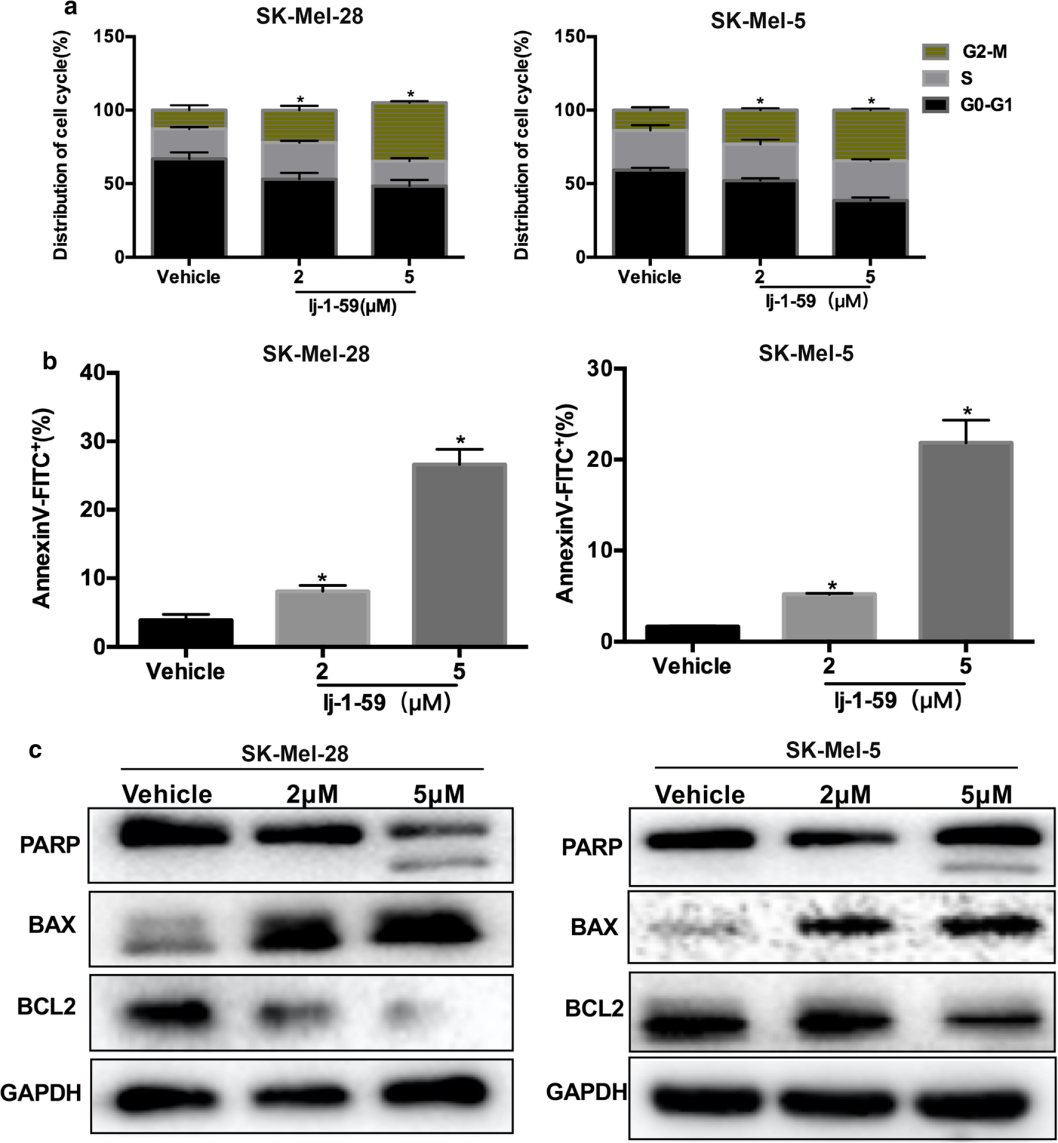
lj-1-59 arrest the cell cycle at G2/M phase and induce apoptosis in melanoma cells. **a** Cell cycle analysis of SK-Mel-28 (left panel) and SK-Mel-5 (right panel) cells with increasing dose lj-1-59 for the 48 h. The cell cycle distribution was detected by flow cytometry as described in “Methods”. The results represent the means (n = 4) ± SD, and asterisk (*) indicates a significant difference (p < 0.05, Chi-square test). **b** Apoptosis analysis of SK-Mel-28 (left panel) and SK-Mel-5 (right panel) cells with increasing dose lj-1-59 for 48 h. The results represent the means (n = 4) ± SD, and asterisk (*) indicates a significant difference (p < 0.05, Student’s t-test). **c** Western Blot analysis of apoptosis-associated proteins in SK-Mel-28 (left panel) and SK-Mel-5 (right panel) cells with lj-1-59 treatment for 48 h

Previous results demonstrated that lj-1-59 suppresses melanoma cell growth in vitro and in vivo. Here, we showed that this compound induced cell cycle arrest at the G2/M phase and promoted apoptosis. As shown in Fig. 3a and Additional file 1: Fig. S2c, the cell cycle was arrested in the G2/M phase after lj-1-59 treatment in melanoma cells. In addition, we found that 5 µM of lj-1-59 treatment induced 21.7%, 27.5% and 38.1% apoptosis in SK-Mel-5, SK-Mel-28 and A375 (Fig. 3b and Additional file 1: Fig. S2d) cells, respectively. Moreover, this compound induced PARP cleavage and increased BAX expression, whereas BCL2 expression was downregulated after lj-1-59 treatment in different melanoma cell lines (Fig. 3c and Additional file 1: Fig. S2e).

### lj-1-59 treatment affects the cell cycle and DNA damage according to RNA-Seq

**Fig. 4:**
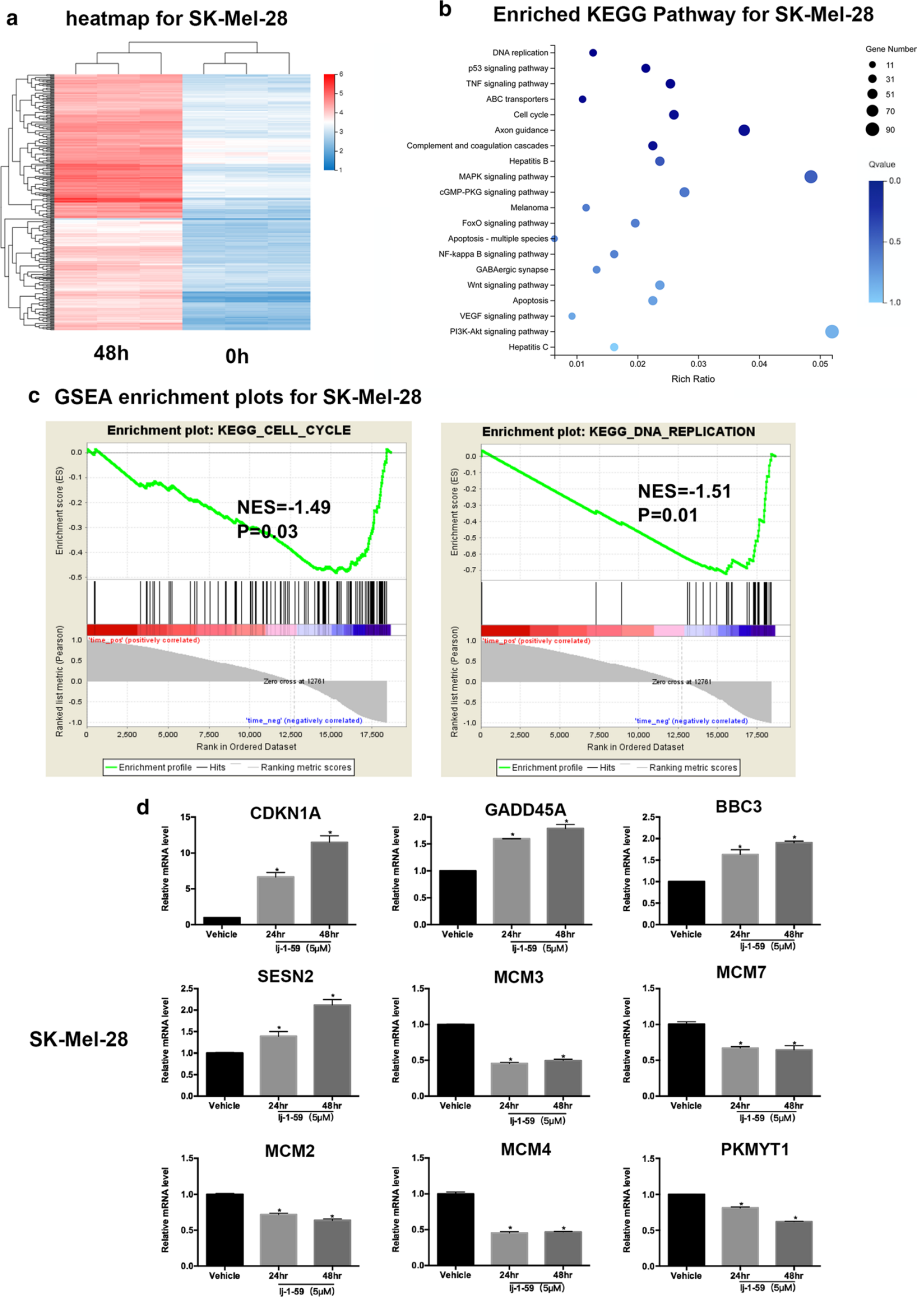
RNA-seq analyses of the effect of lj-1-59 on the gene expression profile. **a** The heatmap of SK-Mel-28 after lj-1-59 treatment. **b** Top 20 enriched KEGG pathways after lj-1-59 treated. **c** GSEA enrichment plots after lj-1-59 treated, and Normalized enrichment score (NES) and Normalized *p*-value (P) are shown in each plot. **d** SK-Mel-28 cells were treated with 5 µM lj-1-59 for 48 h. Then extract total RNA to Q-RT-PCR analysis as described in “Methods”. The results are expressed as the mean (n = 6) ± SD. Significant differences were evaluated using Student’s t-test, and an asterisk (*) indicates a significant difference (*p *< 0.05)

To identify the possible molecular mechanism of lj-1-59 for antitumor activity, we analyzed transcriptional alterations in melanoma cells after lj-1-59 treatment in various melanoma cell lines. The RNA-seq results showed that most genes were up-regulated, whereas other genes were down-regulated after 48 h treatment (Fig. 4a and Additional file 1: Fig. S3a). The top 20 enriched pathways included p53, TNF, FoxO, MAPK, apoptosis and cell cycle pathways (Fig. 4b and Additional file 1: Fig. S3b) according to a KEGG database analysis in differentially expressed genes. Moreover, the GSEA analysis revealed that the effect of lj-1-59 was related to the cell cycle and DNA damage (Fig. 4c and Additional file 1: Fig. S3c), which is consistent with previous results. Based on the above analysis, we hypothesized that cell cycle and DNA damage were pivotal pathways regulated by this compound. Next, we verified key differentially expressed genes, including *P21* (*CDKN1A*), *PUMA* (*BBC3*), *GADD45A, PKMYT1, SESN2, MCM2, MCM3, MCM4* and *MCM7* (Fig. 4d, Additional file 1: Figs. S3d, S4e), which play crucial roles in the cell cycle or DNA damage.

### lj-1-59 treatment induces DNA toxicity by increasing ROS products

**Fig. 5:**
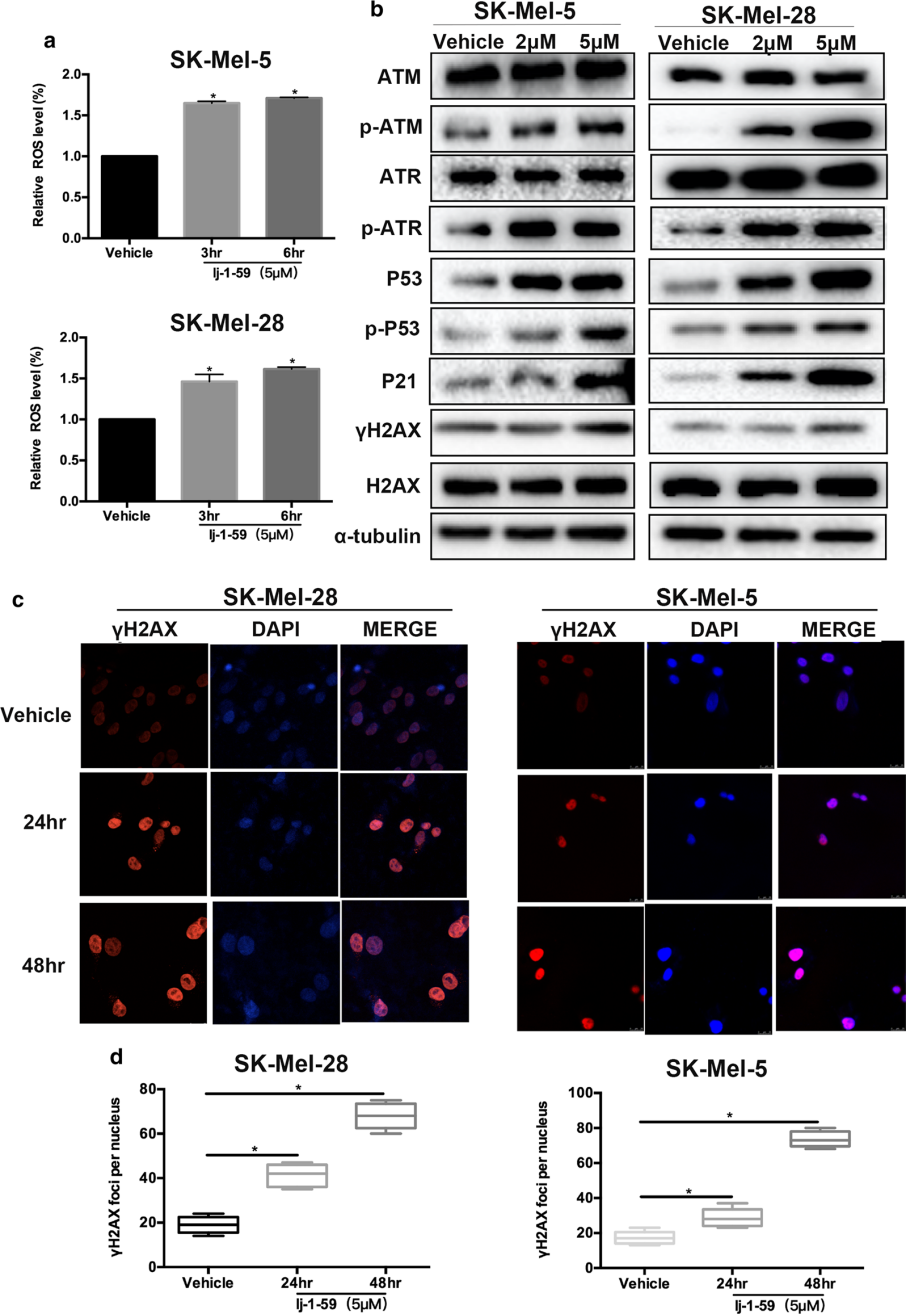
lj-1-59 treatment induces DNA damage by increasing ROS. **a** The level of ROS of SK-Mel-5 (upper panel) and SK-Mel-28 (lower panel) cells were treated with 5 µM lj-1-59 for 0–6 h. **b** Western Blot analysis of cell cycle-associated proteins and DNA damage-associated proteins in SK-Mel-5 (left panel) and SK-Mel-28 (right panel) cells with increasing does lj-1-59 treatment for 48 h. **c**, **d** γH2AX of SK-Mel-28 (left panel) and SK-Mel-5 (right panel) cells were stained by immunofluorescence after 5 µM lj-1-59 treated and calculated. The results in **d** was represent as the mean (n = 6) ± SD, and asterisk (*) indicates a significant difference using Student’s t-test (*p *< 0.05)

ROS are produced by aerobic metabolism, which initiates various biological functions, such as DNA toxicity, cellular death and chronic inflammation [30]. The increase in ROS products has been involved in tumorigenesis and anticancer effects, depending on the level of these products. At a lower level, ROS promote carcinogenesis through the regulation of proliferation, angiogenesis and metastasis, while a high level of ROS induces DNA toxicity, resulting in cellular apoptosis and cell death, which leads to antitumor effects [31]. Unexpectedly, we found that lj-1-59 treatment significantly induced ROS products in melanoma cell lines (Fig. 5a and Additional file 1: Fig. S4a). Additionally, lj-1-59 treatment remarkably activates the ATM/ATR signaling pathway, including p-ATM, p-ATR, γH2AX, P53, p-P53 and P21 (Fig. 5b and Additional file 1: Fig. S4d). ATM or ATR plays critical roles in DNA damage responses and activates downstream molecules, including the p53 pathway, which causes cell cycle arrest at the G2/M phase and apoptosis [32, 33]. γH2AX is a biomarker of DNA damage, and lj-1-59 treatment also increases the accumulation of γH2AX in the nucleus (Fig. 5c, d and Additional file 1: Fig. S4b, c). To investigate the association of ROS generation with lj-1-59-induced cell death, we exposed Sk-Mel-28 cells to lj-1-59 in the absence or presence of NAC (*N*-acetylcysyeine, ROS scavenger). Then we detected anti-apoptotic and anti-arrest cell cycle effect of NAC in lj-1-59-induced cell death by flow cytometry. NAC can significantly reduced lj-1-59-induced cell apoptosis compared with lj-1-59 treatment (Additional file 1: Fig. S5b). In addition, the G2/M phase arrest was reversed partly to control levels in SK-Mel-28 co-treated with NAC (10 mM) and lj-1-59 (Additional file 1: Fig. S5a). These data indicate that ROS play an essential role in the apoptosis and cell cycle induced by lj-1-59 in melanoma cells. Next, We verified the role of NAC in inhibiting ROS generation using flow cytometry. The results show that the levels of ROS can be impeded by NAC (Additional file 1: Fig. S5c). Taken together, these data suggested that lj-1-59 treatment raises ROS products and induces DNA damage and apoptosis in melanoma cells.

### lj-1-59 attenuates BRAFi-resistant melanoma cell growth

**Fig. 6:**
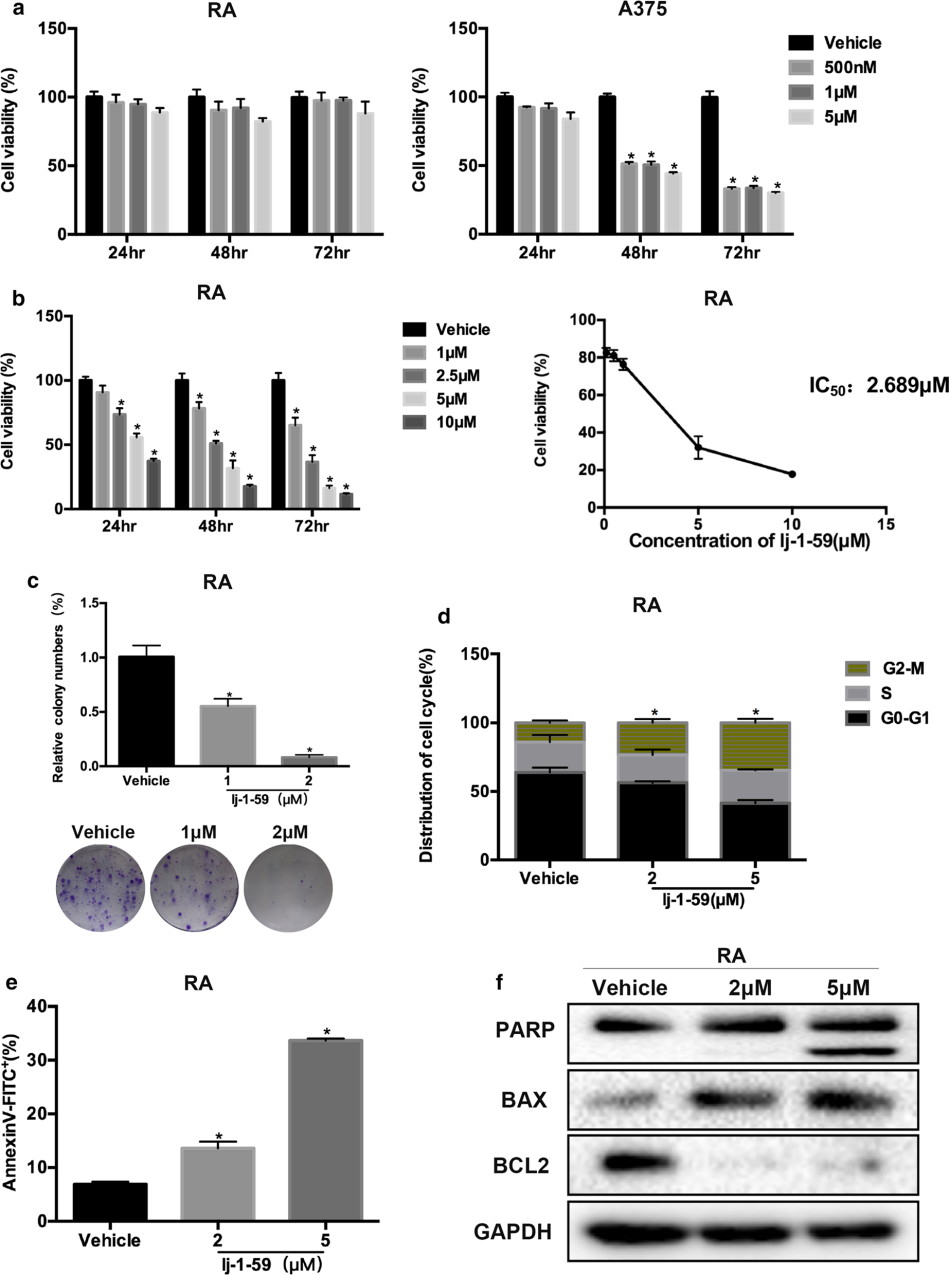
Effect of lj-1-59 on BRAFi-resistant melanoma cells. **a** BRAFi-resistant melanoma cells (RA) were generated as described in “Methods”. RA (left panel) and parental A375 (right panel) cells were prepared in 96-well plates. The cells were treated with PLX4032. Cell viability was determined by CCK-8 assay. The results represent the means (n = 6) ± SD, and asterisk (*) indicates a significant difference (p < 0.05, Student’s t-test). **b** RA cells were treated with increasing dose lj-1-59 for 0-72 h (left panel). Cell viability was determined by CCK-8 assay. The results represent the means (n = 6) ± SD, and asterisk (*) indicates a significant difference (p < 0.05, Student’s t-test). The IC_50_ values of lj-1-59 in RA cells were automatically calculated by GraphPad Prism software (right panel). **c** RA cells were prepared in 6-well plates. The cells were treated with increasing dose lj-1-59 for 24 h. After 2 weeks, the number of colonies was assessed and quantified as described in “Methods”. The results represent the means (n = 5) ± SD, and asterisk (*) indicates a significant difference (p < 0.05, Student’s t-test). **d** Cell cycle analysis of RA cells with increasing dose lj-1-59 for the 48 h. The cell cycle distribution was detected by flow cytometry as described in “Methods”. The results are expressed as the means (n = 4) ± SD, and asterisk (*) indicates a significant difference (p < 0.05, Chi-square). **e** RA cells were treated with increasing dose lj-1-59 for the 48 h. Apoptosis was detected by flow cytometry as described in “Methods”. The results are expressed as the means (n = 4) ± SD, and asterisk (*) indicates a significant difference (p < 0.05, Student’s t-test). **f** Western Blot analysis of apoptosis-associated proteins in RA cells with lj-1-59 treatment for 48 h. GAPDH was used as a loading control

**Fig. 7:**
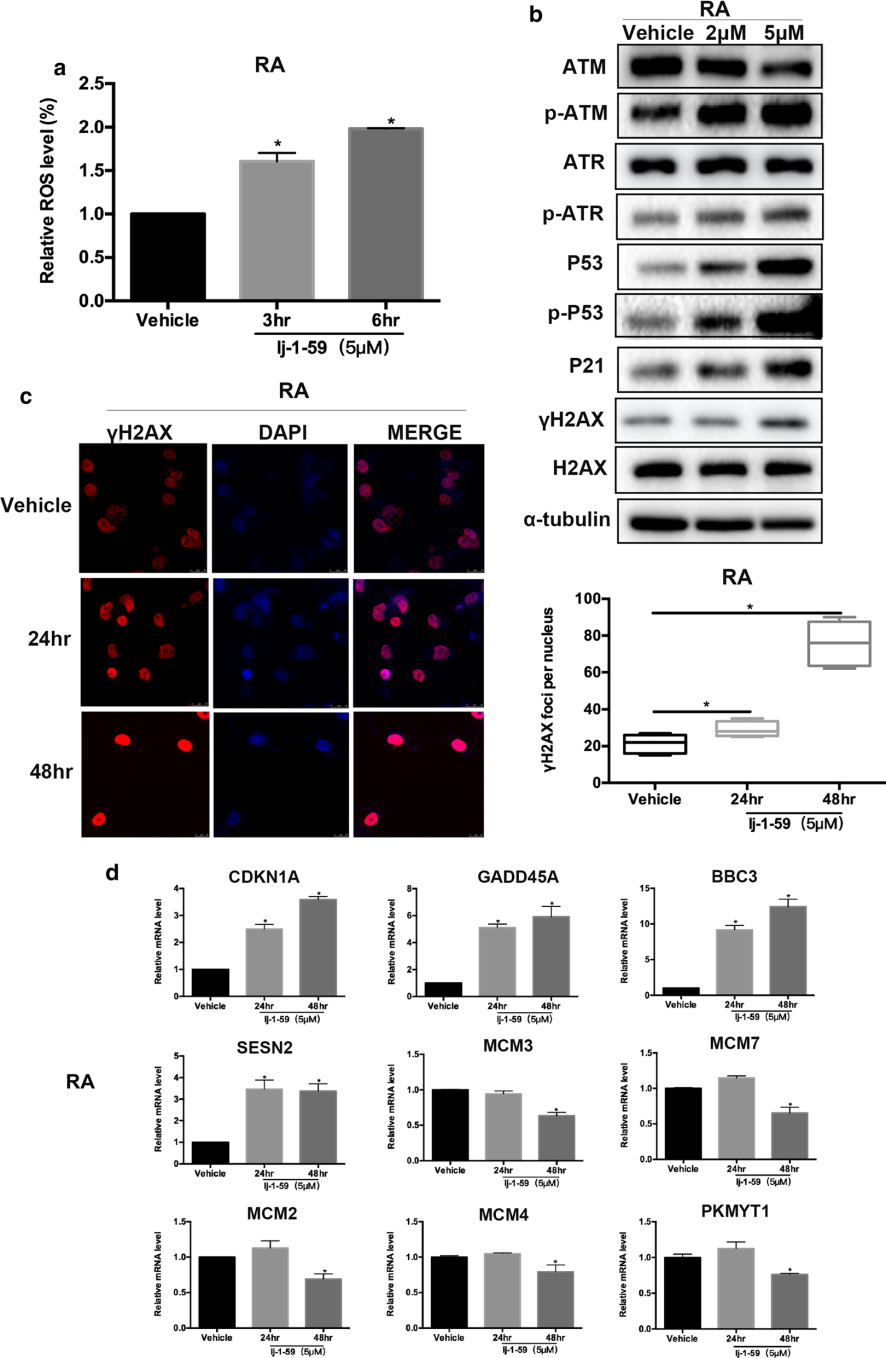
lj-1-59 induces DNA damage by increasing ROS in RA cells. **a** RA cells were treated with 5 μM lj-1-59 for 0-6 h, the level of ROS was measured by flow cytometry. The results are expressed as the means (n = 4) ± SD, and asterisk (*) indicates a significant difference (p < 0.05, Student’s t-test). **b** Western Blot analysis of cell cycle-associated proteins and DNA damage-associated proteins in RA cells with increasing does lj-1-59 treatment for 48 h. α-tubulin was used as a loading control. **c** RA cells were treated with 5 μM for 0–48 h, and γH2AX was stained by immunofluorescence (left panel) and calculated (right panel). The results are expressed as the mean (n = 5) ± SD, and asterisk (*) indicates a significant difference (p < 0.05, Student’s t-test). **d** RA cells were treated with 5 μM lj-1-59 for 48 h. Then extract total RNA to Q-RT-PCR analysis as described in “Methods”. The results are expressed as the mean (n = 5) ± SD. Significant differences were evaluated using Student’s t-test, and an asterisk (*) indicates a significant difference (p < 0.05)

The dysregulation of RAS/MAPK and PI3K/AKT with BRAF mutations in 60% of patients showed that these pathways are key drivers of melanoma development and progression [34, 35]. Although the administration of a BRAF inhibitor (such as vemurafenib) improves patient survival, approximately 70% of patients acquire drug resistance within 6 months [36, 37]; therefore, overcoming drug resistance is a challenge for melanoma-targeting therapeutic treatments. The BRAF-resistant cells (RA) from parental A375 cells were generated as described previously [9, 38] (Fig. 6a). Surprisingly, our finding showed that lj-1-59 treatment dramatically reduced cell viability in a dose- and time-dependent manner in RA cells, and the IC_50_ value for lj-1-59 was 2.69 µM (Fig. 6b), and this compound also inhibited melanoma cell growth on plates (Fig. 6c). Similar to its effects on regular melanoma cells, lj-1-59 treatment causes cell cycle arrest at the G2/M phase (Fig. 6d) and apoptosis (Fig. 6e), including upregulating the expression of cleaved PARP and BAX and decreasing BCL2 expression (Fig. 6f). Consistent with the previous results, NAC can significantly reduced lj-1-59-induced cell apoptosis (Additional file 1: Fig. S5b) and partly reversed the G2/M arrest (Additional file 1: Fig. S5a). lj-1-59 treatment significantly increased ROS products (Fig. 7a), which can be impeded by NAC (Additional file 1: Fig. S5c), leading to DNA toxicity, which increased p-P53, P21, p-ATR, and p-ATM expression (Fig. 7b) and γH2AX foci formation (Fig. 7c). Furthermore, we found that lj-1-59 treatment significantly influences *P21 (CDKN1A), PUMA (BBC3), GADD45A, PKMYT1, SESN2, MCM2, MCM3, MCM4* and *MCM7* expression at the transcriptional level (Fig. 7d), which is consistent with the results in non-BRAFi-resistant melanoma cells, indicating that this compound has antitumor activity for melanoma treatment, regardless of BRAFi resistance.

## Discussion

Natural products and their synthetic analogues are characterized by low cytotoxicity and antitumor activity, which have been a concern for the development of antitumor drugs [39]. Among these natural products, chalcone exhibits diverse biological activities, including antitumor effects [23]; for example, chalcone directly inhibits the activity of IκB kinases (IKKs), which subsequently reduces downstream NF-κB activation, resulting in enhanced apoptosis induced by TNF or chemotherapeutic drugs [40]. Chalcone also inhibits VEGF-induced endothelial cell growth and angiogenesis through the PI3K/AKT signaling pathway in vivo [41].

In this study, we investigated the effects of lj-1-59, a chalcone derivative, on melanoma treatment. Our results demonstrated that lj-1-59 significantly inhibited the growth of melanoma cells, regardless of BRAFi resistance both in vitro (Figs. 1 and 6) and in vivo (Fig. 2). The IC_50_ values for lj-1-59 in SK-Mel-5, SK-Mel-28, and A375 were 1.172 µM, 1.368 µM and 2.002 µM, respectively (Fig. 1c, Additional file 1: Fig. S2a). Moreover, we also found that lj-1-59 treatment induced cell cycle arrest and apoptosis (Fig. 3a–c, Additional file 1: Fig. S2c–e). Although we found that the IC_50_ values of Ij-1-59 to immortalized non-tumorigenic cells is higher than melanoma cells, it is still difficult to calculate therapeutic index of the drug, which is a shortage of this study. In future study, drug toxicology, metabolism and other related experiments will be performed to test the safety of this compound in vivo, which provide more evidences for final clinical administration.

Next, we performed RNA-seq to investigate the effect of lj-1-59 on the signaling pathways. The major pathways, such as DNA replication, P53, cell cycle, and apoptosis, were affected after lj-1-59 treatment (Fig. 4a–c, Additional file 1: Fig. S3a–c). We additionally confirmed the expression with mRNA levels of key genes after treatment with lj-1-59 in melanoma cells, which indicated that *P21, BBC3, SESN2* and *GADD45A* expression were significantly upregulated, while *MCM2, MCM3, MCM4, MCM7* and *PKMYT1* were significantly downregulated (Fig. 4d, Additional file 1: Figs. S3d, S4e). P53 is a tumor suppressor gene, and since its discovery, the inhibitory effect of this molecule on tumor growth has been extensively studied. P53 responds to various types of stress, such as DNA damage and hypoxia, and as a result, this protein plays an important role in supporting cell survival and promoting cell death [42]. P53 protects cells from mild stress damage by eliminating ROS, but ROS accumulation in turn can induce p53-mediated apoptosis in cancer cells [43–45].

P21 plays a key role in cell cycle regulation, which is a well-known targeting gene regulated by p53 in response to various stresses, including DNA damage-induced cell cycle arrest, particularly in the G2/M phase [46–49]. SESN2 is a stress-inducing protein that is also considered a downstream molecule of p53 [50]. GADD45A is a sensor molecule for ROS-induced DNA damage by directly inducing cell cycle arrest and apoptosis [51–54]. MCM2-7 is required for the initiation and elongation steps of DNA replication, which have essential functions in DNA replication [55]. Accumulating evidence has shown that MCM2-7 is significantly overexpressed in various tumors, such as cervical cancer and breast cancer. Moreover, MCM4 and MCM6 expression are clinically relevant to tumor stage [56]. In addition, a novel finding demonstrated that MCM proteins not only regulate S-phase checkpoints but also directly interact with key checkpoint components to regulate DNA repair procedures after DNA damage [57, 58].

The maintenance of genomic stability after DNA toxicity mainly depends on the DNA damage repair system and the cell cycle checkpoint. DNA damage induces the arrest of the cell cycle at the G2/M phase to delay cell cycle progression, ensuring sufficient time to repair damaged DNA [59, 60]. During DNA damage, ATM or ATR activates a variety of downstream pathways, including p53, which leads to cycle arrest or apoptosis [61–63]. In our study, p-ATR, p-ATM, γ-H2AX at Ser139, p-p53 and p53 were upregulated after lj-1-59 treatment (Fig. 5b, Additional file 1: Fig. S4d), indicating that lj-1-59 has DNA toxicity. Interestingly, lj-1-59 treatment significantly increased ROS products (Fig. 5a, Additional file 1: Fig. S4a), and NAC can not only impeded the generation of ROS (Additional file 1: Fig. S5c), but also significantly induced lj-1-59-induced cell apoptosis, partly reversed the G2/M arrest (Additional file 1: Fig. S5a, b). Reactive oxygen species (ROS) are a class of oxygenates that are directly or indirectly converted from molecular oxygen, which has a more active chemical property [64]. It is reported that the generation of ROS in cancer may be due to the reduction of free radical scavenging enzymes or Warburg effect [65]. Although physiologically active oxygen (ROS) levels are necessary to maintain many cellular functions, excessive ROS production could disrupt oxidative balance, leading to cell damage and cell death [66]. Evidence has shown that malignant cells are more susceptible to oxidative stress than normal cells [67]; therefore, a high level of ROS causes DNA damage, which eventually leads to tumor cell necrosis and apoptosis.

## Conclusions

**Fig. 8:**
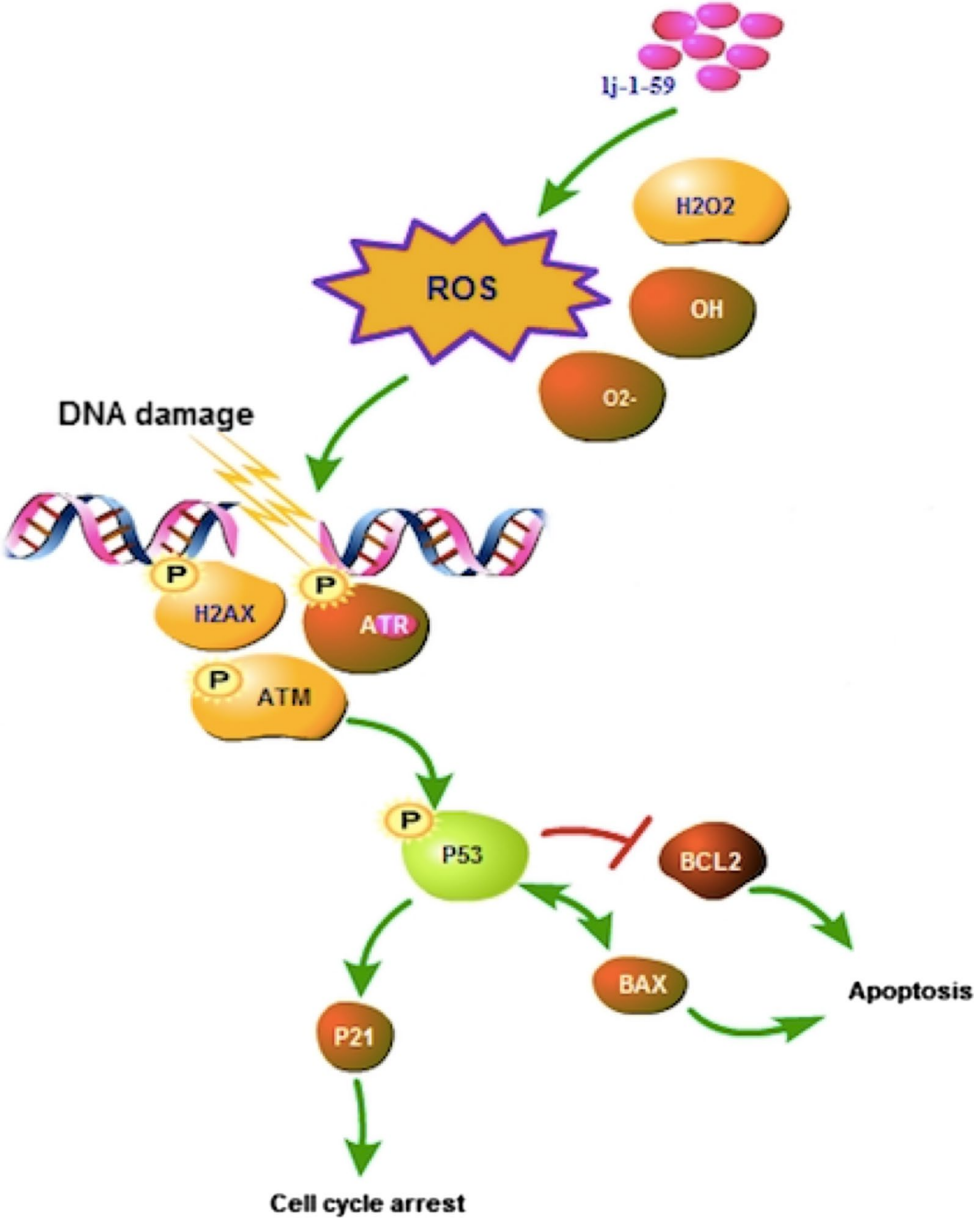
Schematic diagram of the mechanism of lj-1-59. lj-1-59 induces DNA damage by increasing intracellular ROS levels. ATM and ATR are activated after DNA damage, then regulating downstream target protein P53, leading to cell cycle arrest and apoptosis

In this study, we found that lj-1-59 treatment inhibits melanoma cell growth in vitro and in vivo through induced apoptosis and DNA damage by increasing ROS levels (Fig. 8), regardless of BRAFi resistance, suggesting that this compound is a potential therapeutic drug for melanoma treatment.

## Supplementary information

**Additional file 1.** Additional figures.

## Data Availability

The RNA-seq data was uploaded on NCBI (PRJNA545860).

## Abbreviations

CCK-8: Counting Kit-8
BRAFi: BRAF inhibitor
ROS: reactive oxygen species
NGS: next generation sequencing
PI: propidium iodide
NAC: *N*-acetylcysteine
