# Supplementary material for: A novel chalcone derivative has antitumor activity in melanoma by inducing DNA damage through the upregulation of ROS products
Source: Cancer Cell Int. 2020 Jan 30;20:36. doi: 10.1186/s12935-020-1114-5 (PMC6993520; doi:10.1186/s12935-020-1114-5)
Supplement: Supplementary file 1 — Additional file 1. Additional figures. [file 12935_2020_1114_MOESM1_ESM.docx]

**Additional Figures**

**
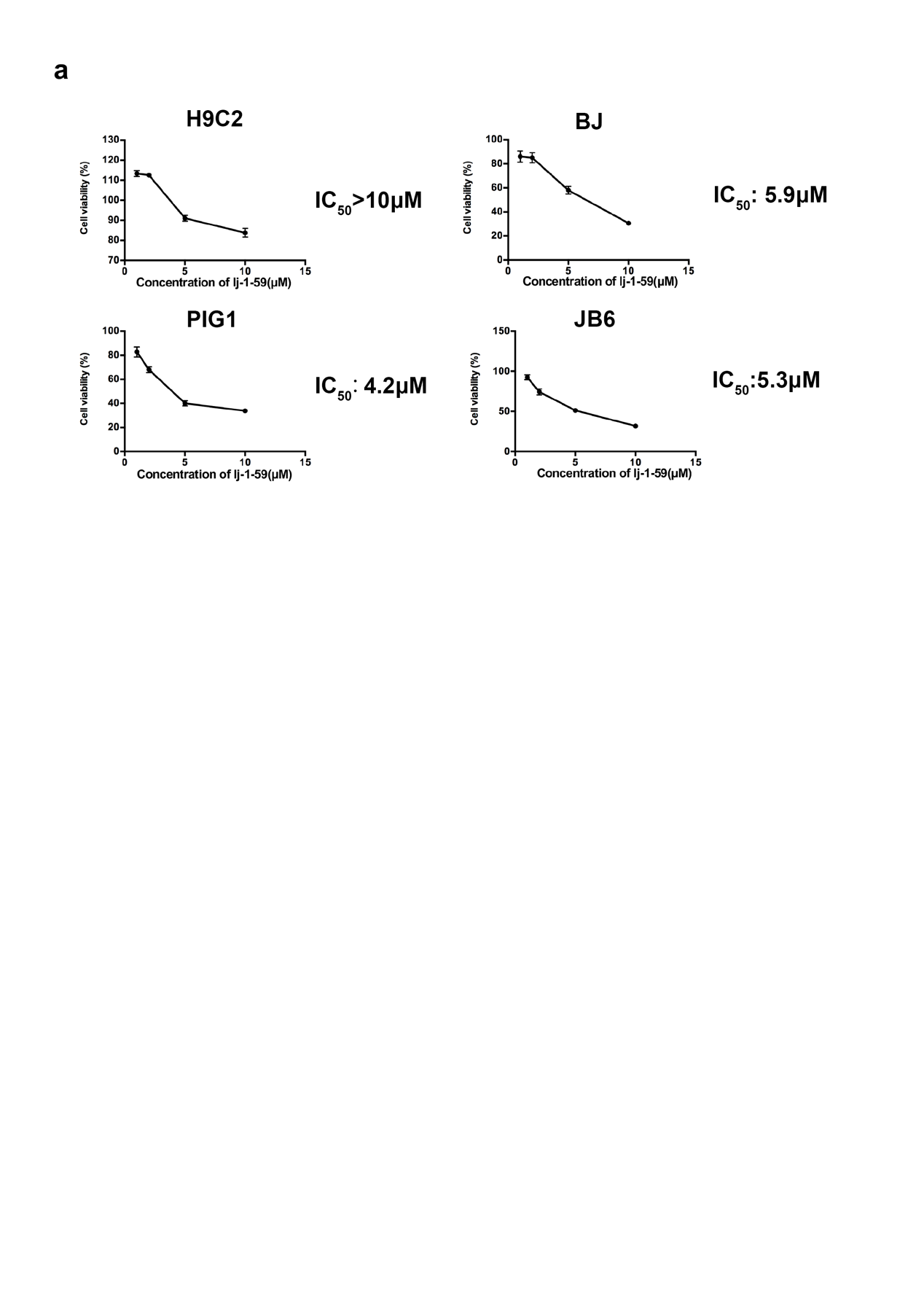
**

**Fig. S1 The effect of lj-1-59 on various normal cells. a** Normal H9C2，PIG1，JB6 and BJ cells were treated with lj-1-59 for different times and dosages as indicated. Cell viability was detected by CCK-8 assay. The results represent the means (n=6) ±S.D.. Significant differences were evaluated using Student’s t-test, and an asterisk (*) indicates a significant difference (p<0.05).

**
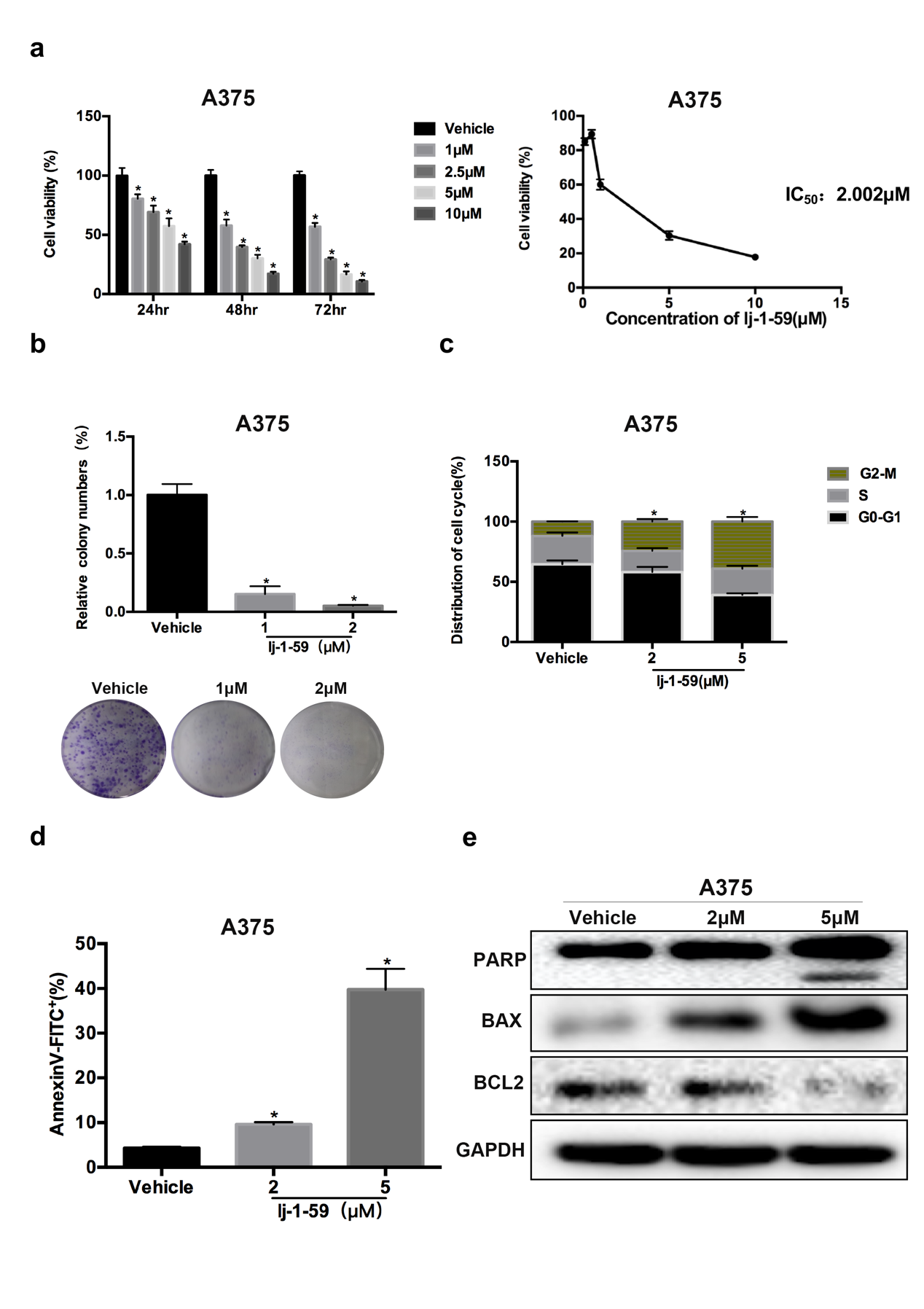
**

**Fig. S2 lj-1-59 arrests the cell cycle at G2/M phase and induces apoptosis in A375 cells. a** Cell viability of A375 cells were determined by CCK-8 assay. The IC_50_ values of lj-1-59 in A375 were automatically calculated by GraphPad Prism software. The results represent the means (n=6) ±S.D.. Significant differences were evaluated using Student’s t-test, and an asterisk (*) indicates a significant difference (p<0.05). **b** A375 cells were prepared in 6-well plates. The cells were treated with increasing dose lj-1-59 for 24h. After two weeks, the number of colonies was assessed and quantified as described in the Materials and Methods. The data represent the mean (n=4) ±S.D., and an asterisk (*) indicates a significant difference (p<0.05, Student’s t-test). **c** Cell cycle analysis of A375 cells with increasing dose lj-1-59 for the 48h. The cell cycle distribution was detected by flow cytometry as described in the Materials and Methods. The results represent the means (n=4) ±S.D., and asterisk (*) indicates a significant difference (p<0.05, Chi-square test). **d** Apoptosis analysis of A375 cells with increasing dose lj-1-59 for 48h. The results represent the means (n=4) ±S.D., and asterisk (*) indicates a significant difference (p<0.05, Student’s t-test). **e** Western Blot analysis of apoptosis-associated proteins in A375 cells with lj-1-59 treatment for 48h.


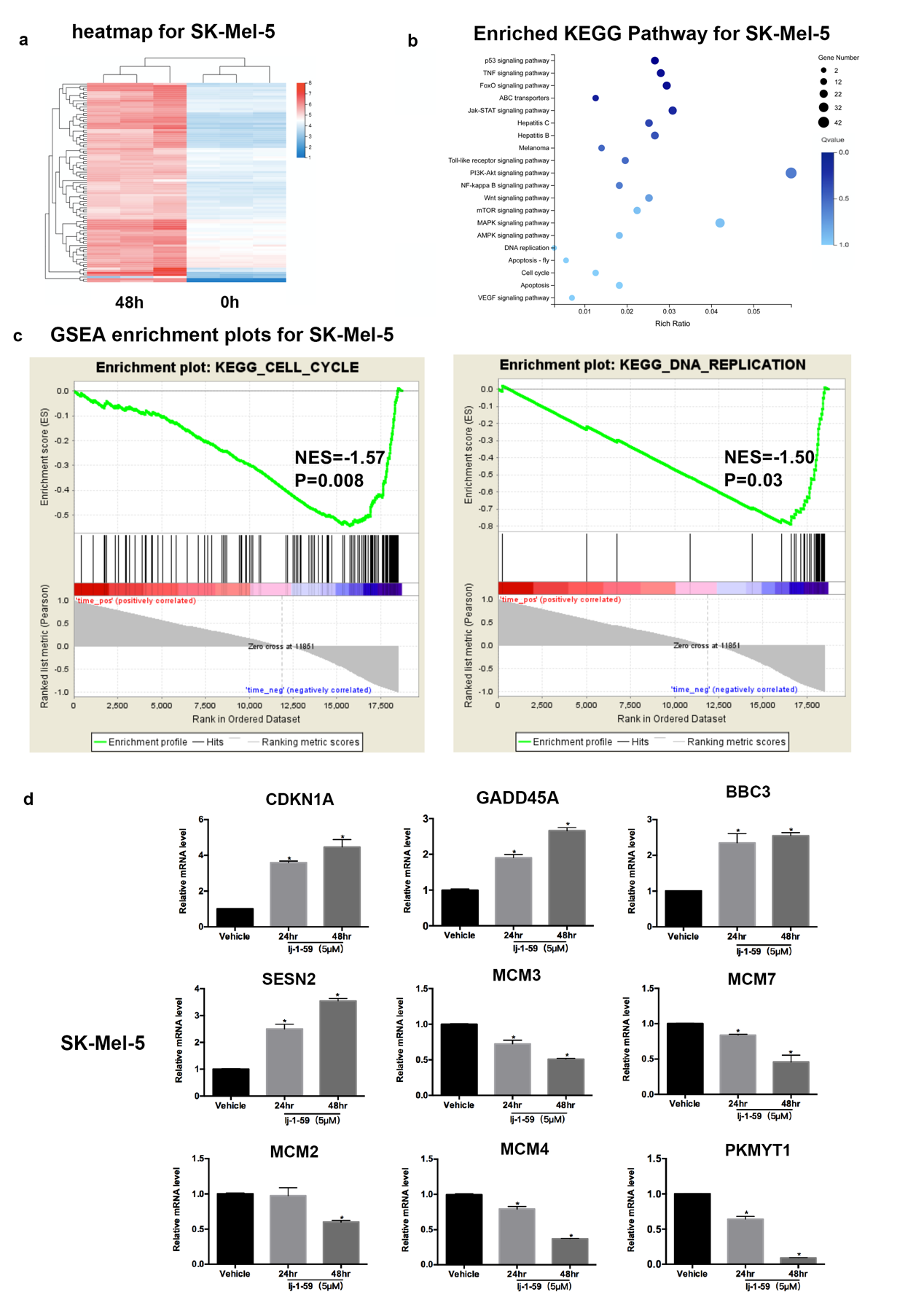


**Fig. S3 RNA-seq analyses of the effect of lj-1-59 on the gene expression profile. a** The heatmap of SK-Mel-5 after lj-1-59 treatment. **b** Top 20 enriched KEGG pathways after lj-1-59 treated . **c** GSEA enrichment plots after lj-1-59 treated, and Normalized enrichment score (NES) and Normalized *p*-value (P) are shown in each plot. **d** SK-Mel-5 cells were treated with 5µM lj-1-59 for 48h. Then extract total RNA to Q-RT-PCR analysis as described in the *Materials and Methods*. The results are expressed as the mean (n=6) ± S.D. Significant differences were evaluated using Student’s t-test, and an asterisk (*) indicates a significant difference(*p*<0.05).


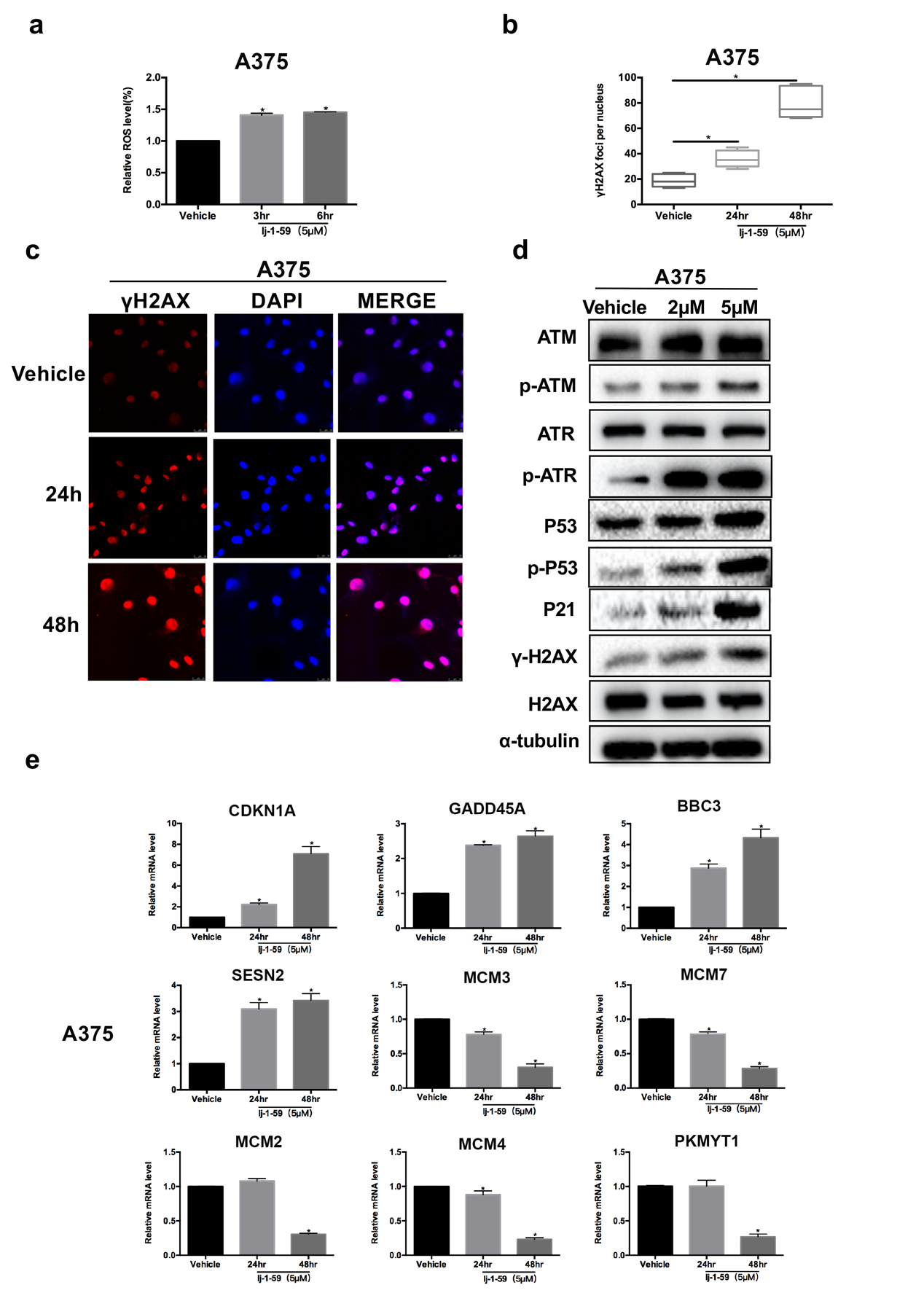


**Fig. S4 lj-1-59 treatment induces DNA damage by increasing ROS in A375 cells. a** The level of ROS of A375 cells were treated with 5µM lj-1-59 for 0-6h. **b, c** γH2AX of A375 cells were stained by immunofluorescence after 5µM lj-1-59 treated and calculated. The results in **b** was represent as the mean (n=6) ± S.D., and asterisk (*) indicates a significant difference using Student’s t-test (*p*<0.05). **d** Western Blot analysis of cell cycle-associated proteins and DNA damage-associated proteins in A375 cells with increasing does lj-1-59 treatment for 48h. **e** A375 cells were treated with 5μM lj-1-59 for 48h. Then extract total RNA to Q-RT-PCR analysis as described in the *Materials and Methods*. The results are expressed as the mean (n=6) ± S.D. Significant differences were evaluated using Student’s t-test, and an asterisk (*) indicates a significant difference(*p*<0.05).


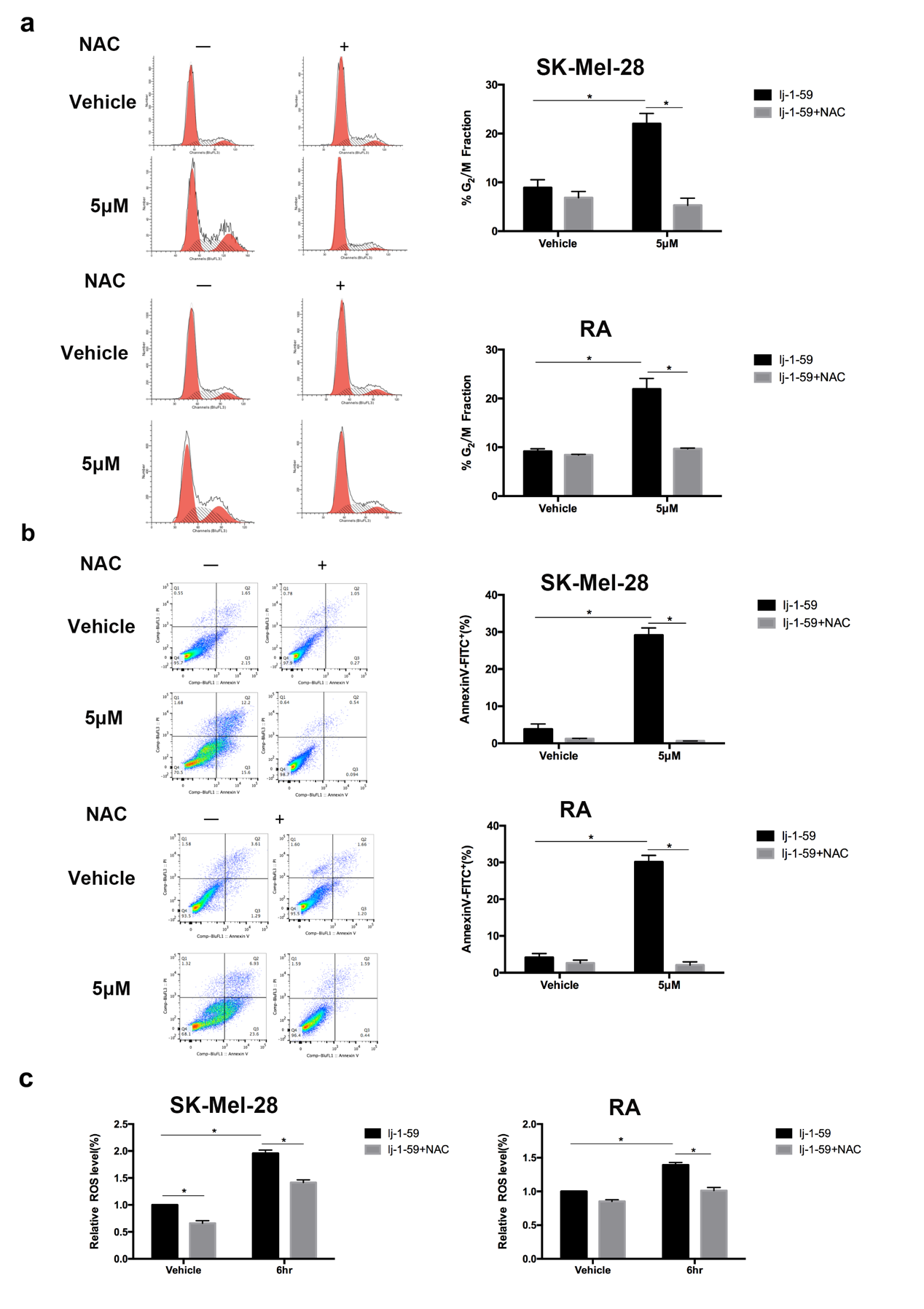


**Fig. S5 NAC can reduced the lj-1-59-induced cell death.** SK-Mel-28 and RA cells with or without NAC (10 mM) were treated with lj-1-59 for 48h. Cells analyzed for cell cycle distribution (**a**) and apoptosis (**b**). **c** SK-Mel-28 and RA cells with or without NAC (5 mM) for 1h were treated with lj-1-59 for 0-6h. The levels of ROS were detected by flow cytometer. The data represent the mean (n=4) ±S.D., and an asterisk (*) indicates a significant difference (p<0.05) using Student’s t-test.
